# Comparative Distribution of Relaxin-3 Inputs and Calcium-Binding Protein-Positive Neurons in Rat Amygdala

**DOI:** 10.3389/fnana.2016.00036

**Published:** 2016-04-07

**Authors:** Fabio N. Santos, Celia W. Pereira, Ana M. Sánchez-Pérez, Marcos Otero-García, Sherie Ma, Andrew L. Gundlach, Francisco E. Olucha-Bordonau

**Affiliations:** ^1^Departamento de Anatomía y Embriología Humana, Facultad de Medicina, Universitat ValenciaValencia, Spain; ^2^Centro de Ciências Biológicas e da Saúde, Universidade TiradentesAracaju, Brazil; ^3^Unitat Predepartamental de Medicina, Universitat Jaume ICastellón, Spain; ^4^The Florey Institute of Neuroscience and Mental HealthParkville, VIC, Australia; ^5^Florey Department of Neuroscience and Mental Health and Department of Anatomy and Neuroscience, The University of MelbourneMelbourne, VIC, Australia

**Keywords:** anxiety, arousal, emotion, motivation, neuropeptide, nucleus incertus, social behavior, theta rhythm

## Abstract

The neural circuits involved in mediating complex behaviors are being rapidly elucidated using various newly developed and powerful anatomical and molecular techniques, providing insights into the neural basis for anxiety disorders, depression, addiction, and dysfunctional social behaviors. Many of these behaviors and associated physiological processes involve the activation of the amygdala in conjunction with cortical and hippocampal circuits. Ascending subcortical projections provide modulatory inputs to the extended amygdala and its related nodes (or “hubs”) within these key circuits. One such input arises from the *nucleus incertus* (NI) in the tegmentum, which sends amino acid- and peptide-containing projections throughout the forebrain. Notably, a distinct population of GABAergic NI neurons expresses the highly-conserved neuropeptide, relaxin-3, and relaxin-3 signaling has been implicated in the modulation of reward/motivation and anxiety- and depressive-like behaviors in rodents via actions within the extended amygdala. Thus, a detailed description of the relaxin-3 innervation of the extended amygdala would provide an anatomical framework for an improved understanding of NI and relaxin-3 modulation of these and other specific amygdala-related functions. Therefore, in this study, we examined the distribution of NI projections and relaxin-3-positive elements (axons/fibers/terminals) within the amygdala, relative to the distribution of neurons expressing the calcium-binding proteins, parvalbumin (PV), calretinin (CR) and/or calbindin. Anterograde tracer injections into the NI revealed a topographic distribution of NI efferents within the amygdala that was near identical to the distribution of relaxin-3-immunoreactive fibers. Highest densities of anterogradely-labeled elements and relaxin-3-immunoreactive fibers were observed in the medial nucleus of the amygdala, medial divisions of the bed nucleus of the stria terminalis (BST) and in the endopiriform nucleus. In contrast, sparse anterogradely-labeled and relaxin-3-immunoreactive fibers were observed in other amygdala nuclei, including the lateral, central and basal nuclei, while the nucleus accumbens lacked any innervation. Using synaptophysin as a synaptic marker, we identified relaxin-3 positive synaptic terminals in the medial amygdala, BST and endopiriform nucleus of amygdala. Our findings demonstrate the existence of topographic NI and relaxin-3-containing projections to specific nuclei of the extended amygdala, consistent with a likely role for this putative integrative arousal system in the regulation of amygdala-dependent social and emotional behaviors.

## Introduction

The amygdala is considered a central node for processing adaptive social and emotional behavior (Aggleton, 1993; Phelps, 2006; Adolphs, 2008). Sensory information arising from cerebral cortex and thalamus enters the amygdala where information acquires emotional value (LeDoux, 2000). In addition, amygdala function extends beyond Pavlovian-like fear conditioning with a role for amygdala processing described in valence (positive and negative affective), reward, decision-making and recognition of emotional facial expressions (Benarroch, 2015). In rodents, olfactory information from the main and accessory olfactory bulbs enters the amygdala where conspecific and allospecifics are recognized and social interactions are evaluated (Scalia and Winans, 1975; Ferguson et al., 2001; Pro-Sistiaga et al., 2007; Trainor et al., 2010). Other telencephalic inputs to the amygdala that originate in prefrontal cortex (McDonald, 1998; Vertes, 2004) and hippocampus (Canteras and Swanson, 1992) shape emotional processing by adding information to past emotional experiences or the context in which emotional experiences takes place (Sierra-Mercado et al., 2011). Intrinsic connections between amygdala subnuclei convey information to amygdala outputs (Pitkänen et al., 1997; Jolkkonen and Pitkänen, 1998; Schmitt et al., 2012). These connections are filtered/modulated by intrinsic GABAergic circuits (Sun and Cassell, 1993) and most GABAergic neurons in the amygdala contain the calcium-binding proteins, parvalbumin (PV), calbindin (CB28kD) or calretinin (CR; McDonald and Mascagni, 2001).

The amygdala is strongly innervated by projections arising from a variety of monoamine neuron populations in the brainstem. For example, serotoninergic afferents are dispersed across multiple regions of the amygdala (Steinbusch, 1981; Smith and Porrino, 2008; Bonn et al., 2013). Arousal and stress induce an increase in serotonin concentrations in the amygdala during fear conditioning (Kawahara et al., 1993; Yokoyama et al., 2005; Mo et al., 2008; Christianson et al., 2010) and serotonin transporter inhibitors (SSRIs) are used to treat amygdala-related disorders, including anxiety, panic and phobias (Sheehan et al., 1993; Gorman, 2003). SSRIs induce increases in serotonin levels in amygdala (Bosker et al., 2001) and increased expression of the serotonin transporter in the amygdala reduces fear (Bocchio et al., 2015). This increased expression of serotonin transporter results in reduction of fear and produces impairment of recruitment of amygdala PV neurons (Bocchio et al., 2015). In contrast, catecholaminergic afferents specifically target the intercalated nuclei (Asan, 1998), which are important for conveying information from the prefrontal cortex to the central nucleus, subserving different aspects of processing extinction memories (Quirk et al., 2003). Dopaminergic cells of the lateral part of the ventral tegmental area and dorsal edge of the substantia nigra pars compacta innervate the amygdala, targeting mainly the intercalated nuclei (Loughlin and Fallon, 1983; Asan, 1998), and dopamine D2 receptor agonist infusion into the amygdala impairs the recall of fear memories (Nader and LeDoux, 1999). In addition, noradrenergic inputs to the amygdala contribute to the reconsolidation of fear memories (Debiec and LeDoux, 2006; Chen and Sara, 2007), and enhancement of noradrenergic transmission impairs extinction of conditioned fear (Debiec et al., 2011). Furthermore, drugs that modify monoaminergic signaling and metabolism have been widely used for many years as treatments of psychiatric disorders, such as anxiety, posttraumatic stress disorders, schizophrenia and autism (e.g., Carlsson and Lindqvist, 1963; Creese et al., 1976; Mailman and Murthy, 2010; Dinnissen et al., 2015). Further in this regard, some studies have reported structural changes in the amygdala in autistic patients. Children with autism have larger amygdala volumes than controls, but during adolescence, volumes become similar in both groups, which indicates an abnormal program of *early* development of the amygdala in autism (Schumann et al., 2004; Schumann and Amaral, 2006).

The *nucleus incertus* (NI) in the pontine tegmentum is another source of afferents to the main amygdala including its extended part (Olucha-Bordonau et al., 2003). Projections from the NI to telencephalic emotional processing centers run in parallel with ascending monoamine pathways from the locus coeruleus, raphe nuclei and the ventral tegmental area (Aston-Jones et al., 1986; Vertes and Kocsis, 1994; Vertes et al., 1999) and target similar areas including the septum, hippocampus, amygdala and prefrontal cortex. The NI is primarily composed of GABAergic neurons (Olucha-Bordonau et al., 2003; Ma et al., 2007), including a population (~30–50%) that expresses the neuropeptide, relaxin-3 (Tanaka et al., 2005; Ma et al., 2007). Relaxin-3 is the ancestral gene/peptide of the relaxin and insulin-like peptide superfamily, and is highly conserved throughout all vertebrate taxa (Wilkinson and Bathgate, 2007). The cognate G_i/o_-protein coupled receptor for relaxin-3 is relaxin family peptide receptor-3, or RXFP3 (also known as GPCR135 and SALPR (Liu et al., 2003; Boels et al., 2004), which is highly expressed within medial and central nuclei of the rat amygdala (Sutton et al., 2004; Ma et al., 2007). Experimental studies in the rat and other species implicate the NI relaxin-3/RXFP3 system in the integrated regulation of arousal/vigilance, stress responses, and associated homeostatic, emotional and cognitive processes (Tanaka et al., 2005; Banerjee et al., 2010; Smith et al., 2011, 2014; Ganella et al., 2013; Ma and Gundlach, 2015).

Acquisition and retrieval of fear memories is reflected by coherent hippocampal and amygdala neuronal activity at theta frequency (4–12 Hz oscillations), also known as theta rhythm (Lesting et al., 2011). Notably, the NI has been demonstrated to modulate hippocampal theta rhythm (Nuñez et al., 2006; Ma et al., 2009), and relaxin-3 neurons in the NI preferentially fire in the early phase of theta oscillations (Ma et al., 2013). It is well established that hippocampal theta rhythm is strongly associated with arousal and exploration (O’Keefe, 1993) and interactions between the hippocampus and the amygdala support the contextual memory components of amygdala-dependent fear conditioning (Sierra-Mercado et al., 2011). In this sense, we have demonstrated that NI lesions result in impaired extinction of conditioned fear (Pereira et al., 2013). Acquisition of fear conditioning was not altered, but NI-lesioned rats exhibited significantly delayed extinction and a stronger freezing response, consistent with disruption of amygdala function and its established regulation of the acquisition and extinction of fear memories (Sierra-Mercado et al., 2011).

The relaxin-3/RXFP3 system has also been linked with modulation of feeding and motivated behaviors. Acute and chronic activation of RXFP3 increases food intake and weight gain in rats (McGowan et al., 2006; Ganella et al., 2013), and the effects of relaxin-3 on feeding behavior are dependent on stress levels and are sex- and species-dependent (Lenglos et al., 2013; Smith et al., 2014). In addition, rats fed with a high-fat diet exhibit differential RXFP3 expression in the medial and central nuclei of the amygdala (Lenglos et al., 2014). Disruption of relaxin-3/RXFP3 signaling impairs motivated food seeking and consumption in mice (Smith et al., 2014); and in an alcohol-preferring (iP) rat strain, icv infusion of the RXFP3 antagonist, R3(B1–22)R, or local infusion of R3(B1–22)R into the *bed nucleus of the stria terminalis* (BST) attenuates stress-induced relapse behavior (Ryan et al., 2013b).

Together, existing data suggest relaxin-3/RXFP3 signaling modulates affective behavior and emotionality, and although the existence of a relaxin-3 innervation of the rat amygdala has been reported (Ma et al., 2007), details of the specific innervation of the various nuclei and subnuclei have not been described. Given the functionally distinct nature of the different amygdala regions (Aggleton, 1993; Davis, 2000; Martínez-García et al., 2012; Olucha-Bordonau et al., 2014), this study aimed to examine the pattern of relaxin-3-immunoreactive afferent fibers, in relation to the delineation of the amygdala nuclei provided by the calcium-binding proteins, PV, CB-28kD and CR (Paxinos et al., 2008).

## Materials and Methods

### Animals

Male Sprague-Dawley rats (300–400 g, *n* = 22) were used in this study. All protocols were approved by the Animal Ethics Committee of the Universitat de València (Spain). All procedures were in line with directive 86/609/EEC of the European Community on the protection of animals used for experimental and other scientific purposes.

### Anterograde Tracer Injections

**Figure 1:**
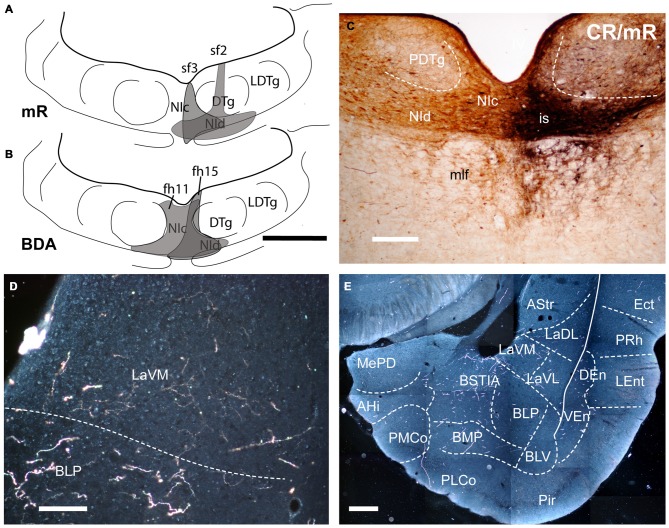
**Injection of an anterograde tracers, into the nucleus incertus (NI) and its transport to the amygdala. (A)** Miniruby (mR) injection sites in the NI of cases sf2 and sf3. **(B)** Biotinylated dextran amine (BDA) injection sites in the NI in cases fh11 and fh15). **(C)** Site of mR injection in the NI of case sf2. **(D)** A darkfield image of the anterograde labeling in the ventromedial subnucleus of the lateral amygdala (LaVM) following a BDA injection into the NI (case fh11). **(E)** A collage of darkfield images of anterogradely-labeled fibers in amygdala at a mid-caudal level. Dense NI projections were present in the intra-amygdala part of the stria terminalis (STIA) and LaVM (case fh11). Calibration bars, 500 μm **(A,B)**, 100 μm **(C)**, 200 μm **(D,E)**.

Rats (*n* = 4) were anesthetized with a combination of ketamine (55 mg/kg i.p., Imalgene, Merial Labs, Barcelona, Spain) and xylacide (20 mg/kg i.p., xilagesic; Lab. Calier, Barcelona, Spain) and trephine holes were drilled in the skull. Anterograde tracer injections into the NI were made using 40 μm I.D. glass micropipettes (coordinates from Bregma: AP −9.6 mm, ML 0 mm and DV 7.4 mm). For anterograde tracing, 15% miniruby (mR, 10 kD biotinylated dextran amine (BDA) rhodamine-labeled; Molecular Probes, Paisley, UK; *n* = 2, Figure 1A) or biotinylated dextran amine (BDA, 10 kD; Molecular Probes, Eugene, OR, USA; *n* = 2, Figure 1B) dissolved in 0.1 M phosphate buffer (PB), pH 7.6 was iontophoretically delivered into the NI by passing a positive current of 1 μA, 2 s on 2 s off through the micropipette, over 10 min. The micropipette was left in place for a further 10 min before withdrawal. After injections, the surgical wound was sutured and rats were injected with Buprex (0.05 mg/kg, i.p., Lab Esteve, Barcelona, Spain) for analgesia. Rats were then allowed to recover for at least 7 days, prior to further processing.

### Brain Fixation and Sectioning

For analysis of tracing studies, IF and ICC, (*n* = 18) rats were deeply anesthetized with Nembutal (150 mg/kg i.p., Euthalender, Barcelona, Spain) and transcardially-perfused with saline (250 ml) followed by fixative (~500 ml of 4% paraformaldehyde in 0.1 M PB, pH 7.4). Brains were dissected and immersed in the same fixative for 4 h at 4°C followed by incubation in 30% sucrose in 0.01 M PBS pH 7.4 for 48 h at 4°C. Coronal sections (40 μm) were collected using a freezing slide microtome (Leica SM2010R, Leica Microsystems, Heidelberg, Germany). For each brain, six series of sections were obtained and collected free-floating in 0.01 M PBS, and stored at −40°C in 30% sucrose in 0.01 M PBS.

### Immunohistochemistry for Relaxin-3 and Calcium-Binding Proteins

For analysis of relaxin-3-immunoreactive fiber distribution in relation to neurochemical/anatomical markers of amygdala nuclei and subnuclei, double-label immunohistochemistry for relaxin-3 and a number of different proteins was conducted. The rabbit relaxin-3 antisera was produced at The Florey Institute of Neuroscience and Mental Health (Parkville, VIC, Australia) and has been characterized and widely used (Ma et al., 2007; Smith et al., 2010; Olucha-Bordonau et al., 2012). In cases where primary antibodies were raised in different host species (rabbit and mouse), a combination of both antibodies in a single primary antibody incubation was used. In cases where primary antibodies were from the same host species, sequential primary and secondary antibody incubations were separated by overnight rinsing. For single primary antibody incubations, sections were rinsed twice in Tris-buffered 0.05 M saline (TBS) pH 8.0 and transferred to blocking solution (4% normal donkey serum (NDS), 2% bovine serum albumin (BSA) and 0.2% Triton X100 in TBS) for 1 h at room temperature. Sections were then transferred to primary antibody solution containing 1:2500 rabbit anti-relaxin-3 (Ma et al., 2007) and either 1:5000 mouse anti-parvalbumin (PV27; Swant 235, Swant, Bellinzona, Switzerland), 1:5000 mouse anti-calbindin-D 28kD (CB38a; Swant), 1:2500 mouse anti-calretinin (CR 6B3; Swant), in TBS containing 2% NDS, 2% BSA and 0.2% Triton X100 for 48 h at 4°C. Relaxin-3 and neuronal markers were sequentially processed in secondary antibody and chromagen visualization.

For relaxin-3, sections were rinsed twice in TBS and incubated in 1:200 biotinylated donkey anti-rabbit antibody (711-065-152, Jackson Immunoresearch, West Grove, PA, USA) for 2 h at room temperature. Sections were then rinsed twice in TBS and transferred to 1:100 Avidin-Biotin-Complex (ABC) (Vectastain, Vector Laboratories, Burlingame, CA, USA). After rinsing (2× TBS) the immunolabeling was revealed as a black reaction product by immersing the sections in 0.025% 3,3′-diaminobenzidine (DAB; Sigma, St Louis, MO, USA), 0.5% ammonium nickel sulfate, 0.0024% H_2_O_2_ in Tris HCl, pH 8.0. Sections were then rinsed for at least 2 h. Amygdala markers were then revealed by incubation in appropriate biotinylated secondary antibody (1:200 biotinylated donkey anti-mouse, Jackson 715-065-150; or 1:200 biotinylated donkey anti-goat, Jackson 705-065-147) for 2 h. Sections were then rinsed twice in TBS and incubated in 1:100 ABC for 1 h. After rinsing (2× TBS) the immunolabeling was revealed as a brown reaction product by incubating the sections in 0.025% DAB 0.0024% H_2_O_2_ in Tris HCl, pH 7.6. Following several rinses in 0.01 M PBS, sections were mounted on chrom alum gelatin-coated slides, air-dried, dehydrated with graded ethanol, cleared with xylene, and coverslipped with DPX (Sigma).

### Immunofluorescence of Relaxin-3 and Calcium-Binding Proteins

In studies of putative contacts between relaxin-3 fibers and neuronal somata and processes positive for calcium binding proteins, a mouse monoclonal relaxin-3 antibody was used in combination with rabbit antisera against calcium binding proteins. The monoclonal antibody against a recombinant relaxin-3 immunogen has been used previously (Tanaka et al., 2005; Ma et al., 2013) and the specificity of the antibody has been demonstrated using brain sections from relaxin-3 gene knockout mice (Watanabe et al., 2011). For double immunofluorescence, sections were rinsed three times in PBS 0.01 M and transferred to blocking solution (4% normal goat serum (NGS), 2% BSA and 0.1% Triton X100 in PBS) for 1 h at room temperature. Sections were then transferred to primary antibody solution containing 1:5 mouse anti-relaxin-3 (cell culture media) and either 1:5000 rabbit anti-parvalbumin (PV27; Swant 235, Swant), 1:10,000 rabbit anti-calbindin-D 28kD (CB38a; Swant), 1:2000 rabbit anti-calretinin (CR 7697; Swant), in PBS containing 2% NGS, 2% BSA and 0.1% Triton X100 for 48 h at 4°C. Relaxin-3 and neuronal markers were sequentially processed for the secondary antibody visualization steps. For relaxin-3, sections were rinsed twice in PBS and incubated in 1:400 goat anti-mouse-Alexa488 antibody (115-545-146, Jackson Immunoresearch) for 2 h at room temperature. Sections were then rinsed three times in PBS and transferred to a new solution for visualizing the calcium binding proteins. Thus, sections were transferred to 1:400 goat anti-rabbit-Cy3 antibody (111-165-003, Jackson Immunoresearch) for 2 h at room temperature. Then sections were rinsed in PBS, mounted on clean slides and coverslipped with Fluoromount^TM^ (F4680, Sigma).

### Immunofluorescence of Relaxin-3 and Synaptophysin

For double-label immunofluorescence, sections were rinsed 2 × 10 min and incubated in blocking media of TBS containing 4% NDS, 2% BSA and 0.1% Triton X-100 for 1 h at room temperature. Sections were then incubated in primary antibody solution containing 1:1250 rabbit anti-relaxin-3 and 1:1000 mouse anti-synaptophysin (Sigma, S5768) in TBS containing 2% NDS, 2% BSA and 0.2% Triton X100 for 48 h at 4°C. Sections were then rinsed (3 × TBS) and incubated in 1:200 Alexa488-conjugated donkey anti-mouse (Jackson Immunoresearch, 715-545-150) and 1:200 Cy3-conjugated donkey anti-rabbit (Jackson Immunoresearch, 711-165-152). Sections were then briefly rinsed in 0.01 M PBS and mounted on chrom alum gelatin-coated slides and coverslipped with Fluoromount^TM^ (F4680, Sigma).

Quantification of colocalization of synaptophysin in relaxin-3 fibers was conducted using sections double-reacted with the monoclonal relaxin-3 antibody and rabbit synaptophysin antisera (ab14692, Abcam, Cambridge, UK). Sections were rinsed three times in PBS 0.01 M and transferred to blocking solution (4% NGS, 2% BSA and 0.1% Triton X100 in PBS) for 1 h at room temperature. Sections were then transferred to primary antibody solution containing 1:5 mouse anti-relaxin-3 and 1:400 rabbit anti-synaptophysin. For relaxin-3 visualization, sections were rinsed twice in PBS and incubated in 1:400 goat anti-mouse-Alexa488 antibody (Jackson Immunoresearch, 115-545-146) for 2 h at room temperature. For synaptophysin visualization, sections were rinsed 3× PBS and transferred to a solution containing 1:400 goat anti-rabbit-Cy3 antibody (Jackson Immunoresearch, 111-165-003) for 2 h at room temperature. Then sections were rinsed in PBS, mounted on clean slides and coverslipped with Fluoromount^TM^ (F4680, Sigma).

### Immunohistochemistry Analyses

DAB immunohistochemistry was examined and recorded using a Nikon Eclipse E600 microscope equipped with a DMX2000 digital camera (Nikon, Tokyo, Japan). Maps of antigen distribution were constructed using a camera lucida tube attached to a Zeiss Axioskop microscope (Zeiss, Munich, Germany) at 20× magnification. These were scanned and reduced to the final size for reproduction. Confocal immunofluorescence was analyzed with a laser confocal scan unit TCS-SP8 equipped with argon and helio-neon laser beams attached to a DMIRB inverted microscope (Leica Microsystems). For Cy3 fluorophore, excitation was 433 nm for 560–618 nm emission. For Alexa 488, excitation was 488 nm for 510–570 nm emission. Serial 0.3 μm sections were imaged to assess colocalization of relaxin-3-immunoreactive terminals with synaptophysin, using Leica Confocal Software (V 2.61).

In *n* = 3 cases, the co-localization of synaptophysin and relaxin-3 was analyzed in different amygdala divisions. Captured images using the 40× objective were taken through a total depth of 10 μm and then reconstructed with the Leica application suite (LAS-X) software. Firstly, we studied the percentage of relaxin-3 fibers that does not co-localize with synaptophysin. Second, we studied the density of synaptophysin boutons along the length of relaxin-3 fibers.

## Results

The delineation of the different amygdala nuclei has been recently documented (Olucha-Bordonau et al., 2014; Paxinos and Watson, 2014) and based on these topographical descriptions, in this study we have considered three regions: the temporal amygdala (TA), the bed nucleus of the stria terminalis (ST^1^) which corresponded to the dorsal extension, and the sublenticular extended amygdala (SLEA), which corresponded to the ventral extension. These divisions are based on topography to facilitate anatomical description, but do not correspond to functional or ontogenetic divisions. Notably, the lateral, basal and cortical nuclei are of pallial origin (Table 1), while the two extensions, together with the medial and central nuclei, constitute the medial and central extended amygdala, which are of sub-pallial origin (Table 2). We have used this hierarchy to analyze the occurrence of anterograde tracing and relaxin-3-immunoreactivity in different nuclei of the amygdala.

**Table 1:** **Distribution of anterogradely-labeled nucleus incertus (NI) fibers and relaxin-3-positive fibers in pallial nuclei of amygdala**.

	Nucleus/area	Anterogradely-labeled NI fibers	Relaxin-3 IR
Cortical pallial	**Lateropallial**		
	Nucleus of the lateral olfactory tract (LOT)	−	−
	Bed nucleus of the accessory olfactory tract (BAOT)	−	−
	Cortex amygdala transition zone (CxA)	−	−
	Amygdalopiriform transition area (APir)	−	−
	Dorsal endopiriform (DEn)	+++	+++
	Ventral endopiriform (VEn)	+++	+++
	**Ventropallial**		
	Anterior cortical amygdaloid nucleus (ACo)	−	−
	Posterolateral cortical amygdaloid nucleus (PLCo)	−	−
	Posteromedial cortical amygdaloid nucleus (PMCo)	−	−
Deep pallial	**Lateropallial**		
	Basolateral (BL)		
	Basolateral anterior nucleus (BLA)	+	+
	Basolateral posterior nucleus (BLP)	++	++
	Basolateral ventral nucleus (BLV)	+	+
	Basomedial		
	Basomedial anterior (BMA)	+++	+++
	Basomedial posterior (BMP)	+	+
	**Ventropallial**		
	Lateral (La)		
	Lateral dorsolateral part (LaDL)	+	+
	Lateral ventrolateral part (LaVL)	++	++
	Lateral medial part (LaM)	++	++
	Amygdalohippocampal transition area		
	Posteromedial (AHiPM)	++++	++++
	Anterolateral (AHiAL)	++++	++++

**Table 2:** **Distribution of anterogradely-labeled NI fibers and relaxin-3 positive fibers in subpallial nuclei of amygdala**.

Nucleus/area	Anterogradely- labeled NI fibers	Relaxin-3 IR
Medial nucleus (Me)		
Medial nucleus anterodorsal part (MeAD)	++++	+++
Medial nucleus anteroventral part (MeAV)	++++	+++
Medial nucleus posterodorsal part (MePD)	+	+
Medial nucleus posteroventral part (MePV)	++	++
Central nucleus (Ce)		
Central nucleus medial division (CeM)	+	+
Central nucleus lateral division (CeL)	+	+
Central nucleus capsular division (CeC)	+	+
Amygdalostriatal transition area (ASt)	+	+
Anterior amygdaloid area (AA)	+++	+++
Intercalated nuclei (I)	++	++
Bed nucleus of the stria terminalis (ST)		
Intraamygdala part (STIA)	+++	+++
Supracapsular part (STSC)	+	+
Medial anterior (STMA)	+++	++
Medial ventral (STMV)	+++	++
Lateral division (STL)	−	−
Lateral dorsal (STLD)	−	−
Lateral intermediate (STLI)	−	−
Lateral juxtacapsular (STLJ)	+	++
Lateral posterior (STLP)	−	−
Fusiform nucleus (Fu)	−	−
Parastriatal nucleus (PS)	−	−
Bed nucleus of the anterior commissure (BAC)	−	−
Sublenticular extended amygdala (SLEA)	+	+
Sublenticular substantia innominata (SI)	−	−
Interstitial nucleus of the	+	+
posterior limb of the anterior		
commissure (IPAC)		

### Nucleus Incertus Projections to Amygdala

**Figure 2:**
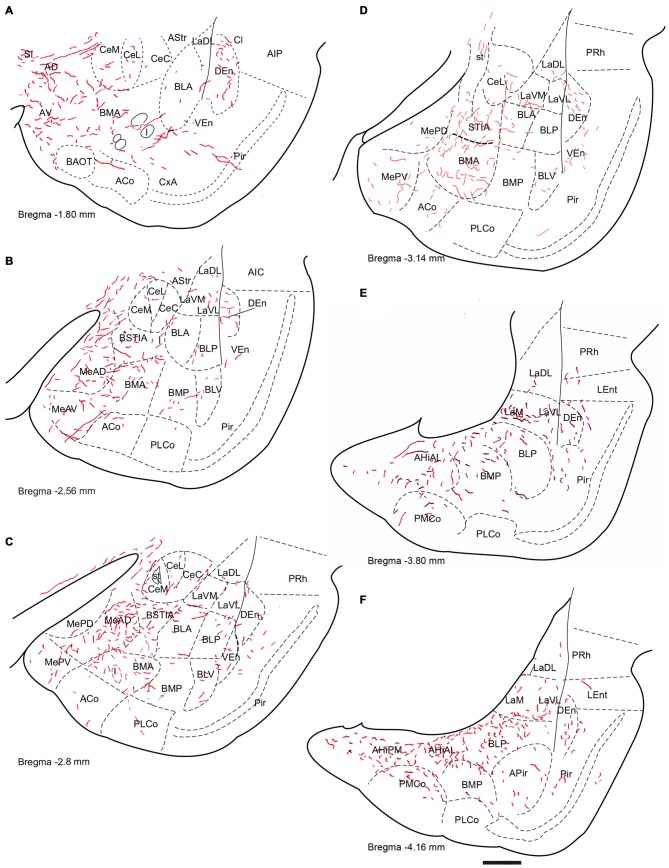
**Distribution of anterograde fiber labeling in the temporal amygdala (TA) from a case with a BDA injection into the NI.** Labeled fibers occurred mainly in the medial aspects of the themporal amygdala and in a broad band in the basal nuclei. **(A–F)** Represents camera lucida drawings of the corresponding bregma levels. Calibration bar 500 μm.

**Figure 3:**
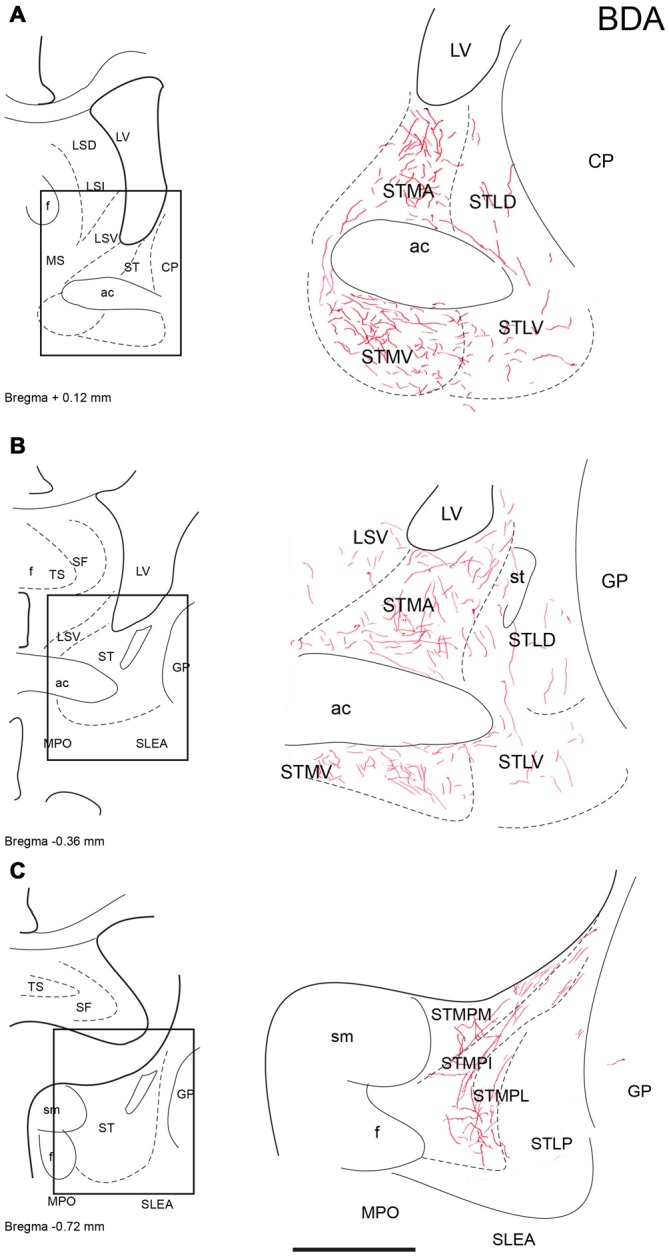
**Distribution of anterograde fiber labeling in the bed nuclei of the stria terminalis (ST) after a BDA injection into the NI.** Labeled fibers mainly occurred in the medial nuclei. **(A–C)** Represents the levels that are shown in a camera lucida detail of the corresponding bregma levels. Calibration bar 250 μm.

**Figure 4:**
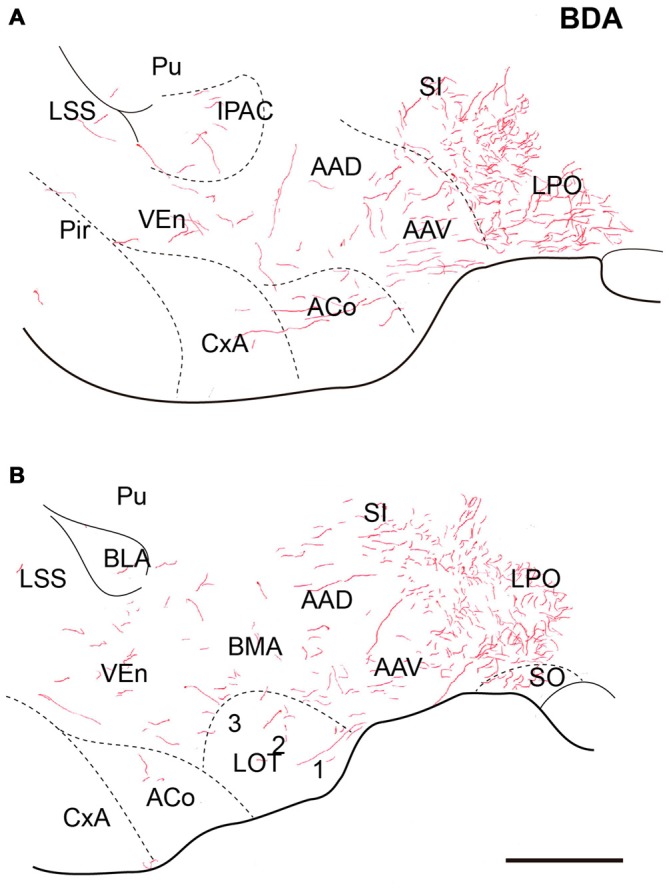
**Distribution of anterograde fiber labeling in the sublenticular extended amygdala (SLEA) after a BDA injection into the NI.** Labeled fibers mainly occurred in the medial aspects of the SLEA. **(A)** Corresponds to Bregma −0–72, **(B)** Corresponds to Bregma −0.84 mm. Calibration bar 250 μm.

We successfully made BDA (*n* = 2) and mR (*n* = 2) tracer injections that were restricted to the NI, with no spread of tracer to the adjacent dorsal tegmental nucleus or pontine raphe nucleus (Figures 1A–C). Anterogradely-labeled fibers in the amygdala detected with both tracers were thin and varicose (Figures 1D,E; data not shown), primarily occupying medial aspects of the TA (Figures 1D,E, 2), and medial aspects of ST and SLEA (Figures 3, 4).

#### Temporal Amygdala (TA)

Anterogradely-labeled fibers entering the amygdala complex appeared to originate from fiber tracts within the medial forebrain bundle and sublenticular area at rostral levels of the TA (Figure 2A). The densest areas of NI innervation occupied the medial amygdala nuclei and a broad band between the basal lateral and cortical nuclei, which corresponded most closely to the basomedial nuclei. Labeled fibers were observed within the four nuclei of the medial amygdala—medial anterodorsal (MeAD), medial anteroventral (MeAV), medial posterodorsal (MePD) and medial posteroventral (MePV; Figures 2B,C). However, in the MePD, the most medial region was devoid of labeled fibers, which were concentrated in the lateral aspects of this nucleus (Figure 2D). Most cortical amygdala nuclei were devoid of labeled fibers except for the inner parts. In contrast, a high density of fibers was observed in the anterior part of the basomedial nucleus (BMA; Figures 2A–D), whereas the posterior part (BMP) contained sparse anterograde labeling. The intra-amygdala part of the ST (STIA) contained a high density of labeled fibers (Figures 2B–D), with a differential distribution observed within the lateral and basolateral nuclei, along the rostrocaudal axis. While these nuclei contained sparse labeling at anterior levels, fibers were more concentrated at caudal levels, forming dense plexuses in the basolateral posterior nucleus (BLP), lateral medial part (LaM) and lateral ventrolateral part (LaVL) of the ventropallidal amygdala (Figures 2E,F). At these caudal levels, a high density of fibers was also observed in the anterolateral (AHiAL) and posteromedial (AHiPM) parts of the amygdalohippocampal transition area (Figure 2F). Sparse labeling was observed in the posteromedial cortical amygdala nucleus (PMCo), and the amygdalopiriform transition area (APir) was devoid of any innervation.

Anterogradely-labeled fibers (BDA and mR) were observed crossing the basomedial nuclei to innervate lateral aspects of the basolateral ventral nucleus (BLV) and the endopiriform nuclei, giving rise to dense plexuses in both the DEn and ventral endopiriform nuclei (VEn; Figures 2B–D, Table 1).

#### Nuclei of the Stria Terminalis (ST)

In the ST, anterogradely-labeled fibers were primarily observed in the medial aspects at all rostrocaudal levels (Figures 3A–C). At rostral and middle levels, fibers were observed in the anterior (STMA) and ventral (STMV) nuclei of the medial nucleus of the ST, encircling the anterior commissure bundles (Figures 3A,C), and dispersed fibers were also observed in the lateral nuclei of the ST. At caudal levels, labeled plexuses were observed in the posterior medial (STMPM), posterior intermediate (STMPI) and posterior lateral (STMPL) parts of the medial nucleus of the ST (Figure 3C). No labeling was observed in any lateral nuclei at these caudal levels.

#### Sublenticular Extended Amygdala

At anterior levels of the TA, a dense plexus of labeled fibers was observed in the lateral preoptic area (LPO) and horizontal limb of the diagonal band. In addition, some fibers ran laterally to the anterior amygdala nuclei. Some fibers were observed in the ventral (AAV) and dorsal (AAD) divisions of the anterior amygdala nuclei, where fibers ran rostrally and dorsally to innervate the sublenticular substantia innominata (Figures 4A,B). Disperse fibers were also observed in the basomedial nuclei of the amygdala and the interstitial nucleus of the posterior horn of the anterior commissure (IPAC; Figures 4A,B).

### Distribution of Relaxin-3 Fibers within the Amygdala

**Figure 5:**
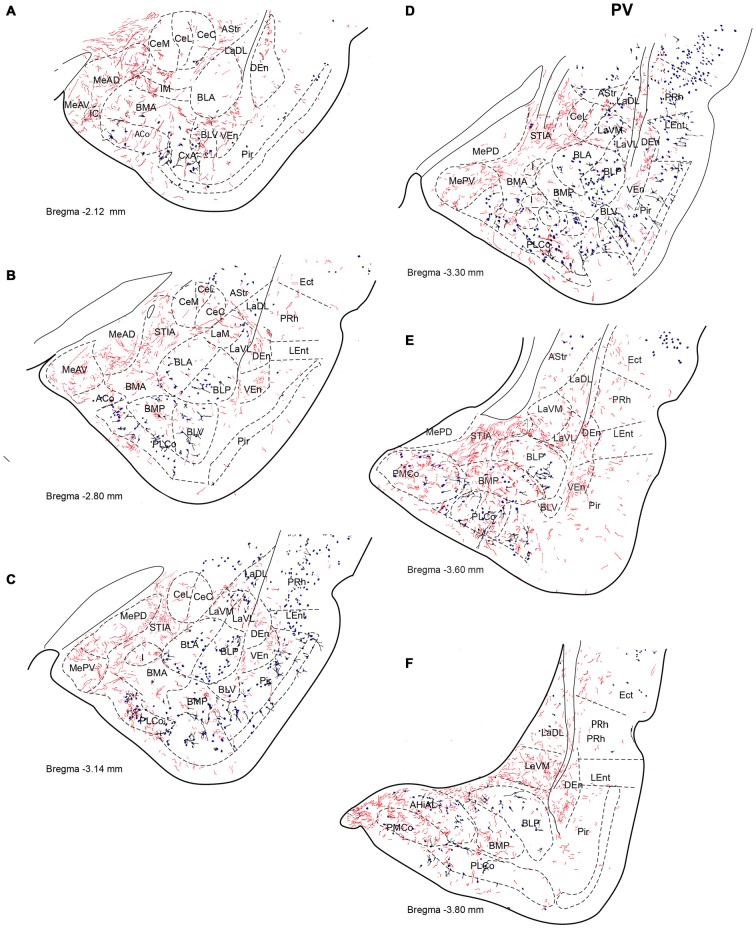
**Distribution of relaxin-3-positive fibers in the TA relative to the distribution of parvalbumin (PV)-positive neurons.** Relaxin-3 fibers located in the medial amygdala areas containing a lower proportion of PV neurons. **(A–F)** Camera lucida drawings of the corresponding Bregma levels. Calibration bar 250 μm.

**Figure 6:**
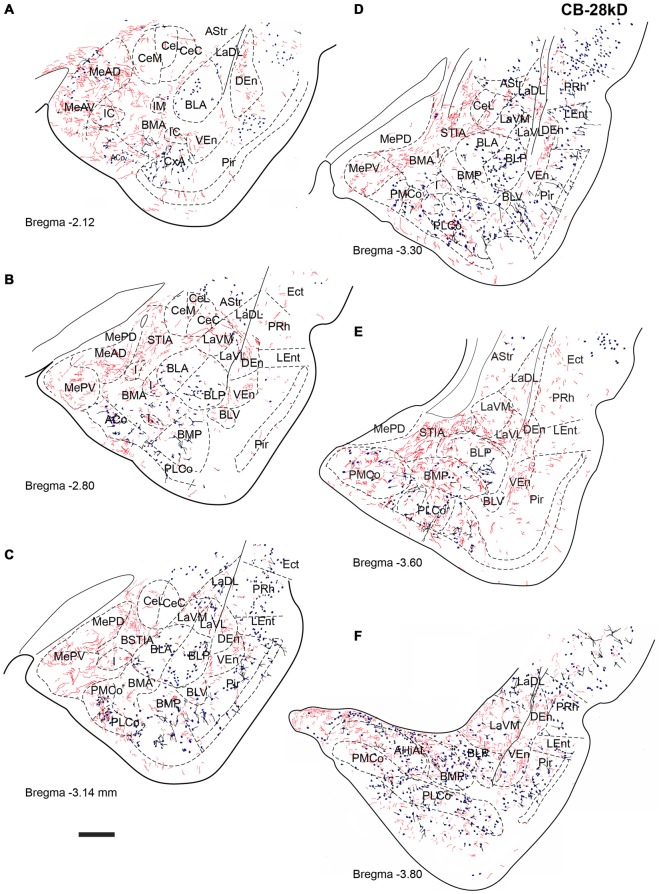
**Distribution of relaxin-3-positive fibers in the TA relative to the distribution of CB-28kD-positive neurons.** Relaxin-3 fibers located in the medial amygdala areas. **(A–F)** Camera lucida drawings of the corresponding Bregma levels. Calibration bar 250 μm.

**Figure 7:**
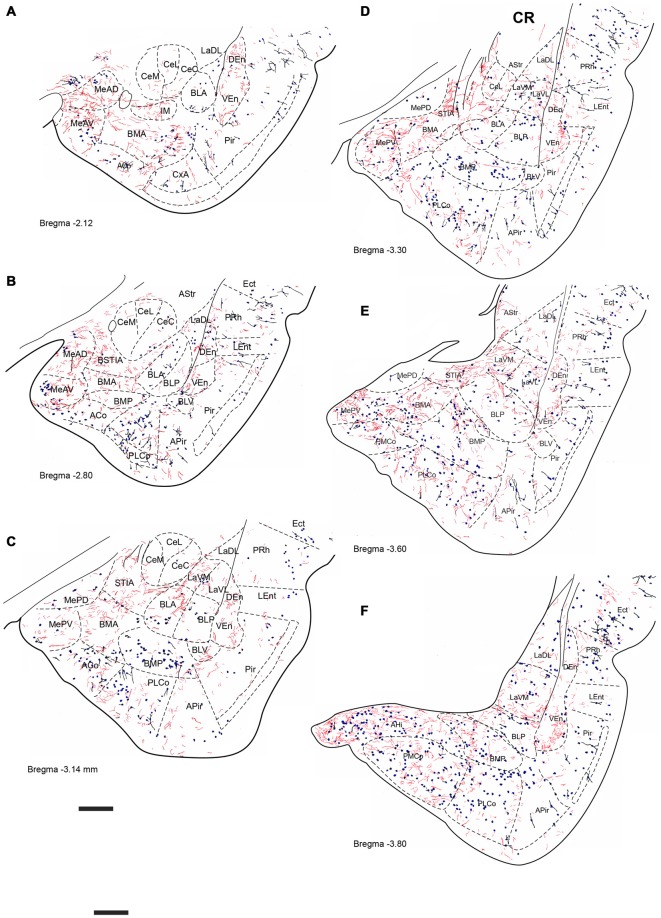
**Distribution of relaxin-3-positive fibers in the TA relative to the distribution of calretinin (CR)-positive neurons.** Relaxin-3 fibers located in the medial and caudal amygdala areas. **(A–F)** Camera lucida drawings of the corresponding Bregma levels. Calibration bar 250 μm.

The distribution of relaxin-3-immunopositive fibers was similar to that observed with anterograde-tracer labeling of NI inputs to the amygdala. In order to identify the nature of target neurons within the different amygdala nuclei, we used double-label immunofluorescence for relaxin-3 and the calcium-binding proteins, PV, calbindin-28kD (CB-28kD) and CR (Figures 5, 6, 7).

#### Relaxin-3 Innervation of Temporal Amygdala (TA)

Six rostrocaudal levels of the amygdala, from −2.2 mm to −3.6 mm relative to Bregma (Paxinos and Watson, 2014), were assessed in these studies. In an effort to better describe the distribution pattern observed, amygdala nuclei and subnuclei were delineated using the distribution of PV, CB-28kD and CR immunoreactivity. In general, PV-, CB-28kD and CR-positive cells appeared dispersed in the amygdala areas derived from a cortical-pallial origin, thus representing good landmarks for the boundary between pallium and subpallium.

Strong PV labeling was present in the neuropil of the BLA and BLP nuclei (data not shown), which clearly differentiates them from the La and the BMA/BMP. A dense concentration of PV-positive neurons occurred in the CxA and in the BLV (Figure 5A). PV cells were abundant in the PLCo (Figures 5B–F). At some levels, PV-positive neurons appeared at the boundary between the La and the BL nuclei (Figure 5E). There were also abundant PV neurons in the AHi (Figure 5F), whereas PV neurons were absent from the medial and central nuclei.

CB-28kD-positive neurons followed a similar pattern, but some CB-28kD neurons were also observed in amygdala areas of subcortical-subpallial origin. This was especially evident in the MeAD and MeAV vs. MePD and MePV. Thus, the occurrence of CB28kD-positive neurons is a specific feature of the anterior nuclei of the medial amygdala (Figure 6A). At more caudal levels, CB28kD neurons were concentrated in the cortical nuclei, including the PLCo and the PMCo (Figures 6C–F).

CR immunoreactivity was present in an intense band in the superficial layer of the cortical and medial amygdala nuclei. CR-positive neurons were also present in the anterior nuclei of the medial amygdala (MeAD and MeAV; Figures 7A–C), but were scarce in the posterior nuclei (MePD and MePV). Only dispersed CR-positive cells were observed in the BMP and BMA (Figures 7B–E), while abundant CR-positive neurons were present in the AHi and in the PMCo (Figure 7F). At these caudal levels CR neurons were concentrated at the boundary between the ventromedial subnucleus of the lateral amygdala (LaVM) and BLP, an area that also contained dense plexuses of RLN3 fibers (Figure 7F).

At the most rostral level, dense plexuses of varicose relaxin-3-positive fibers were observed penetrating the MeAD and MeAV of medial amygdala, originating from fiber tracts in the nearby medial forebrain bundle (Figures 5A, 6A, 7A), which then dispersed within more anterior levels giving rise to dispersed labeling in the AD and AV nuclei and extending to the SLEA. Sparse relaxin-3-positive fibers were also observed innervating the intercalated nuclei surrounding the lateral and basal nuclei, whereas the central nucleus was devoid of immunoreactivity (Figures 5A–C, 6A–C, 7A–C). Dense plexuses of relaxin-3 varicose fibers were observed in DEn and VEn. A moderate relaxin-3 innervation was also observed in a region between the cortical and the basolateral amygdala that corresponded to the basomedial nuclei (Figures 5A,B).

At progressively more caudal levels (Figures 5C,D, 6C,D, 7C,D), the densest relaxin-3 innervation was observed in the anterodorsal (ADMeA) and anteroventral (AVMeA) medial amygdala nuclei. Dense immunoreactivity was also observed in the STIA (Figures 5D,E, 6D,E, 7D,E). Labeling was sparse in the olfactory amygdala and piriform cortex, basal, lateral and central amygdala, and dispersed fibers were observed associated with intercalated cell groups lying between the basolateral and basomedial amygdala nuclei.

At caudal levels, the relaxin-3 innervation was concentrated in inner amygdala nuclei comprising the posteromedial cortical area (PMCo), the AHi and the posterior basomedial nucleus (BMP). A band of intense labeling was also observed at the boundary between the lateral (LaVM) and posterior basolateral nuclei BLP (Figures 5F, 6F, 7F).

#### Relaxin-3 Innervation of the Bed Nucleus of the Stria Terminalis (ST)

**Figure 8:**
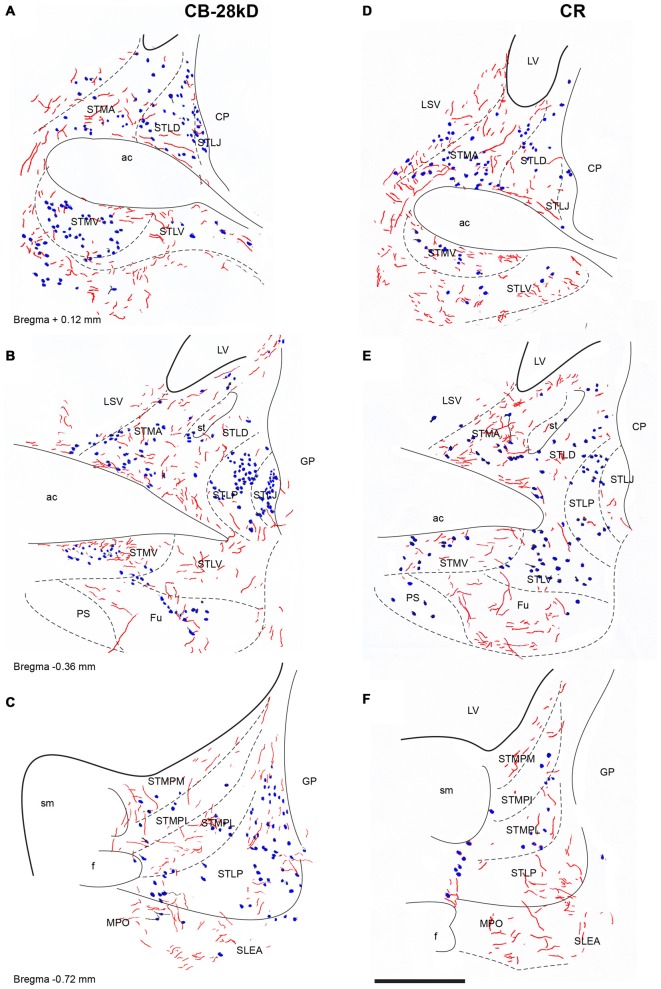
**Distribution of relaxin-3-positive fibers in the nuclei of the ST relative to the distribution of CB-28kD-positive neurons (A,C,E) and CR-positive neurons (B,D,F).** Calibration bar 250 μm.

ST nuclei were well delineated by CB-28kD and CR immunoreactivity and relaxin-3 inputs were primarily confined to the medial aspects of the ST (Figure 8). At the most rostral levels of ST (Figures 8A,D), dense CB-28kD-positive neurons were observed in the lateral nuclei comprising the laterodorsal (STLD) and lateral juxtacapsular nuclei (STLJ), which contained dispersed relaxin-3-positive fibers (Figure 8A). In contrast, CR neurons were abundant in the medial anterior nucleus (STMA), which contained moderate levels of relaxin-3-positive fibers that coursed around the anterior horn of the anterior commissure (Figure 8B). Relaxin-3 inputs were also observed in nuclei adjacent to the anterior commissure bundles, namely the ventral medial nucleus (STMV), whilst the ventral aspects of the lateral nucleus (STLV) contained sparse relaxin-3-positive fibers (Figures 8A,B).

At mid-levels of the ST (Figures 8B,E), few relaxin-3-positive fibers were observed in the medial regions, which comprised both the anterior (STMA) and ventral (STMV) divisions that were clearly identified by a dense cluster of CR-positive neurons (Figure 8D). In contrast, dispersed fibers were observed in the lateral nuclei (STLD, STLP and STLJ), and parastriatal (PS) and funicular (Fu) nuclei.

At caudal levels (Figures 8C,F), relaxin-3-immunoreactive fibers were concentrated in all medial nuclei, comprising the posteromedial (STMPM), posterointermediate (STMPI) and posterolateral (STMPL) divisions, and at this level, some relaxin-3-positive fibers were also found in the posterior division of the lateral nucleus (STLP). At caudal and ventral levels of the ST, relaxin-3 fibers appeared to be in continuity ventrally with labeled fibers coming from the rostral and dorsal aspects of the SLEA (Figures 8C,F).

#### Relaxin-3 Innervation of the Bed Nucleus of the Sublenticular Extended Amygdala (SLEA)

**Figure 9:**
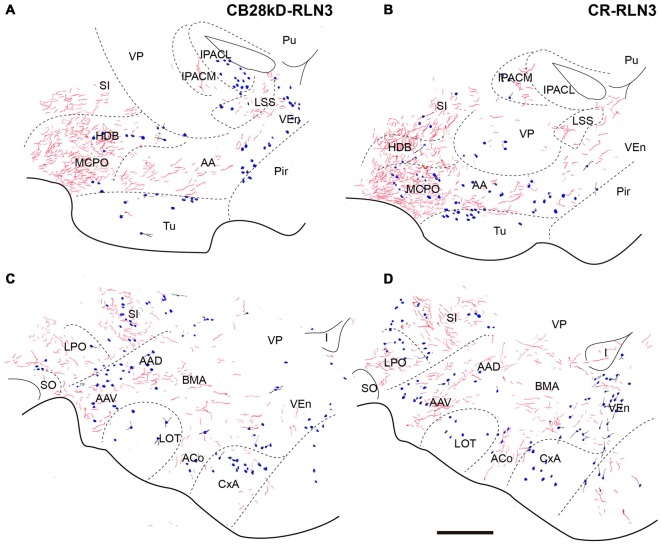
**Distribution of relaxin-3-positive fibers in the SLEA relative to the distribution of CB28kD-positive neurons (A,C) and CR-positive neurons (B,D).** Calibration bar 250 μm.

Relaxin-3-positive fibers were sparse in this rostral extension of the amygdala. At the coronal level of the rostral amygdala, relaxin-3-labeled fibers were most concentrated in the horizontal diagonal band (HDB) and in the magnocellular preoptic nucleus (MPO; Figures 9A,C). From this region, some fibers coursed dorsally into the substantia innominata and laterally forming a band in the anterior amygdala area located between the ventral pallidum and the olfactory tubercle, which contained CB-28kD-positive neurons (Figure 9A), but was devoid of CR-positive cells (Figure 9C). Relaxin-3-labeled fibers were also observed in the VEn and in the medial division of the IPAC, whereas the ventral pallidum lacked relaxin-3-immunoreactivity.

At more caudal levels of the SLEA (Figures 9B,D), a dense plexus of relaxin-3-immunoreactive fibers was observed in the LPO. These fibers extended laterally to the ventral (AAV) and dorsal (AAD) divisions of the anterior amygdala and dorsally to the substantia innominata (Figures 9B,D). Sparse fibers were also observed in the DEn and VEn. The interstitial nucleus of the posterior arm of the anterior commissure contained two divisions, the medial (IPACM) and the lateral (IPACL). While the IPACM contained a dense plexus of relaxin-3 fibers, the IPACL was devoid of fibers, but contained a population of CB-28kD-positive neurons (Figures 9A–C). The ventral pallidum, nucleus of the lateral olfactory tract (LOT) and the bed nucleus of the accessory olfactory tract (BAOT) were devoid of relaxin-3 immunoreactivity (Figures 9B,D).

#### Distribution of mR-Positive NI and Relaxin-3-Positive Inputs and Calcium-Binding Protein-Positive Neurons in the Amygdala

**Figure 10:**
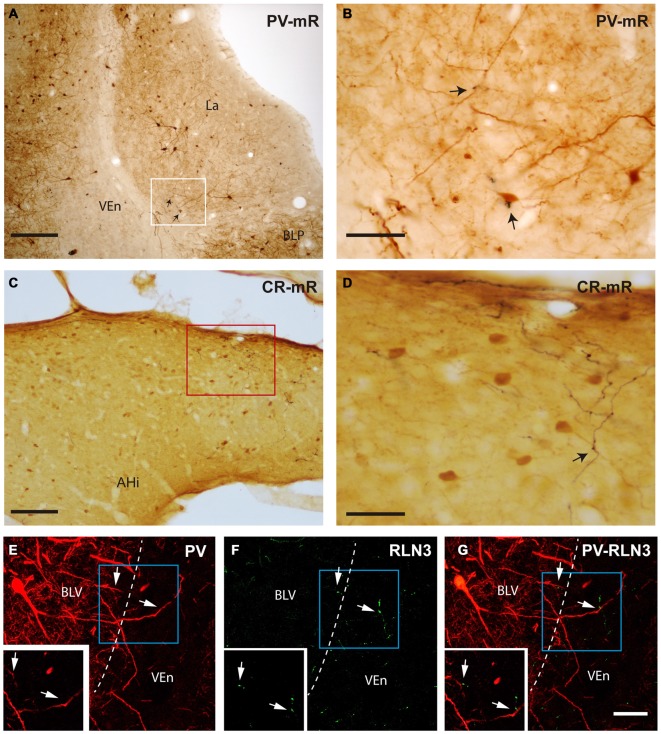
**Close apposition between mR anterogradely-labeled NI fibers or relaxin-3-positive fibers and PV-positive or CR-positive neurons. (A)** Posterior lateral amygdala region (containing mR-positive elements and neurons stained for PV (case sf2). **(B)** Higher magnification of the area labeled in **(A)** representing a close contact between an anterogradely labeled fiber (black) and the soma of a PV-positive neuron. **(C)** Anterograde labeling was abundant in the caudal aspects of the amygdala, particularly in the amygdalohippocampal transition area. **(D)** Some of the anterogradely-labeled elements contacted CR neuron processes (arrow; case sf3). **(E–G)** Confocal images illustrating contacts between relaxin-3 fibers and PV dendrites in the basolateral ventral nucleus (BLV). The main images represent the maximal projection and the insets a single 0.3 μm stack. Some dendrites of these neurons extend beyond the boundaries of the nucleus, in this case in the ventral endopiriform nucleus (VEn) which contained dense relaxin-3 labeling. Calibration bars, 200 μm **(A)**, 20 μm **(B)**, 100 μm **(C)**, 40 μm **(D)**, 30 μm **(E–G)**.

**Figure 11:**
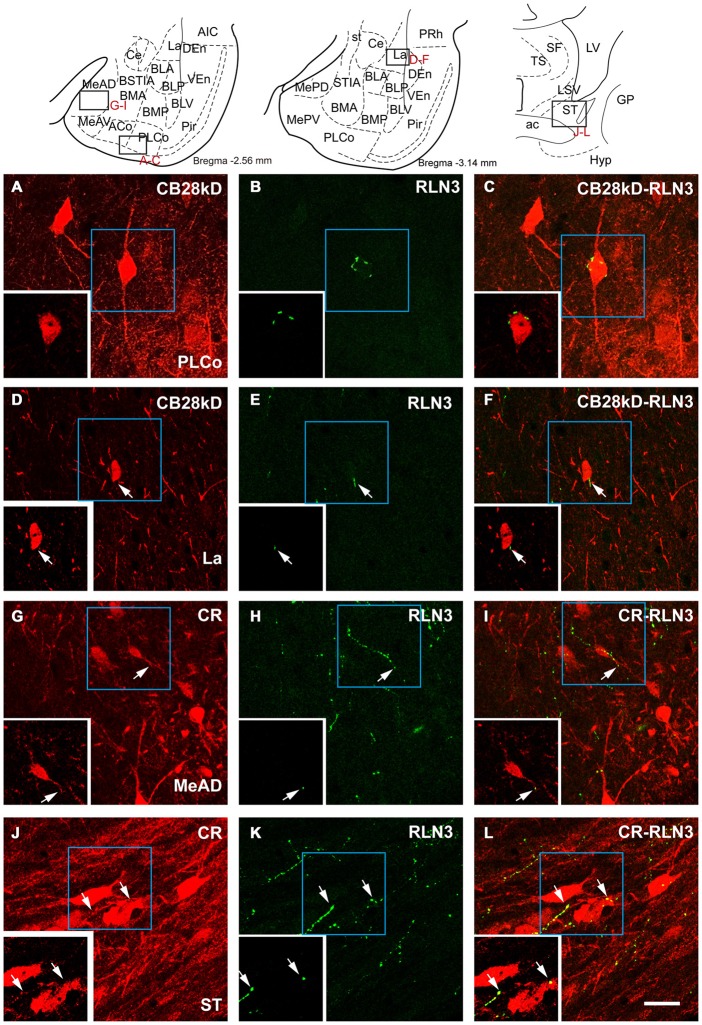
**Confocal images of close apposition between calcium binding positive neurons and processes and relaxin-3 positive fibers.** Large images correspond to a maximal projection of stacks of 20 images of scans of 0.3 μm. Insets illustrate a single 0.3 μm section illustrating the contact. **(A–C)** Contact between a relaxin-3 fiber and a CB28kD somata in PLCo. **(D–F)** Contact of a relaxin-3 fiber with a CB28kD fiber in the La. **(G–I)** Contacts between RLN3 fibers and CR positive somata and processes in the MeAD. **(J–L)** Contacts between RLN3 fibers and CR somata and processes in the STM. Calibration bar 20 μm.

Close appositions were observed between anterogradely-labeled fibers positive for mR and PV soma and processes in the ventrolateral division of the lateral amygdala (Figures 10A,B). In permanent DAB stained samples, it was also possible to find close contacts of mR positive fibers and CR processes in the AHi (Figures 10C,D). Confocal images from different amygdala nuclei revealed a graded pattern of contacts between relaxin-3-positive fibers and calcium binding protein somata and processes. These contacts were evident in the La and BL divisions and in some cases the contacts occurred between fibers and processes outside the boundaries of the nucleus (Figures 10E–G). In the PLCo relaxin-3 fibers were found surrounding the neuronal somata of CB-28kD-positive neurons (Figures 11A–C). Also relaxin-3 fibers contacted somata of CB-28kD neurons in the lateral nucleus (Figures 11D–F). CR neurons of the medial amygdala also received contacts from relaxin-3-positive fibers (Figures 11G–I). Finally, contacts were also observed in the medial nucleus of the ST where relaxin-3 fibers contacted processes (Figures 11J–L).

### Co-Localization of Relaxin-3 and Synaptophysin

**Figure 12:**
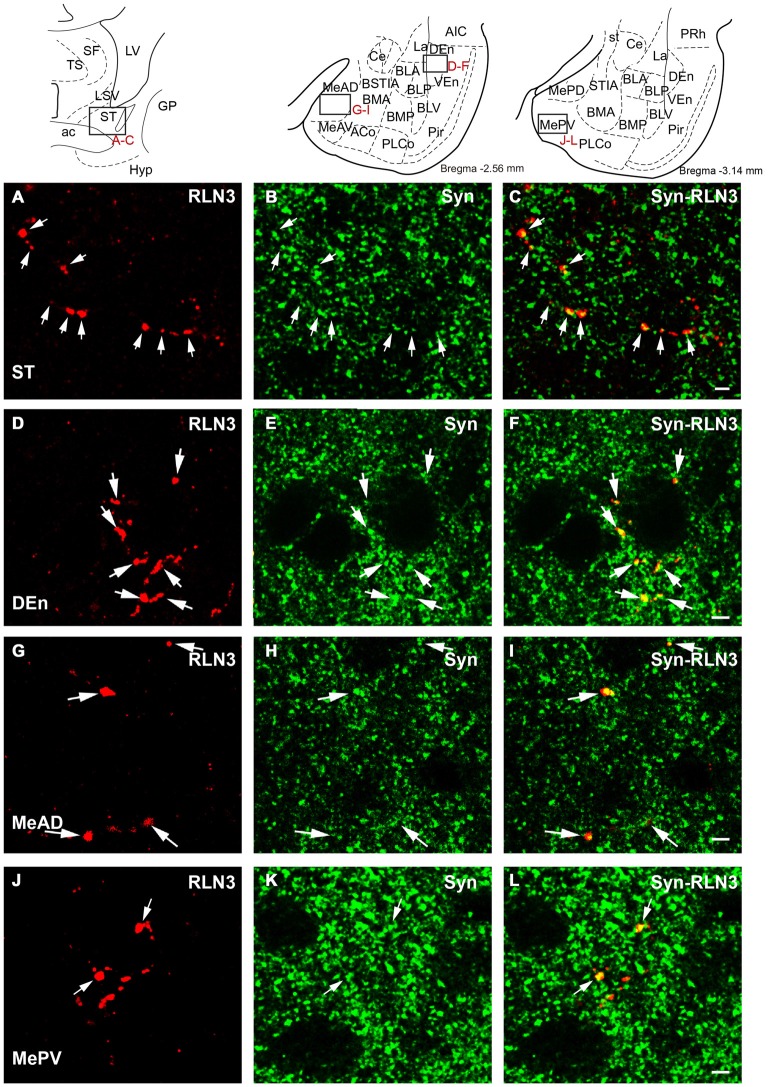
**Confocal images revealing the colocalization of relaxin-3 (red) and synaptophysin (green) in the framed areas of uppermost schematic images.** Single 0.5 μm sections displaying the localization (arrows) of relaxin-3 **(A)** synaptophysin **(B)** and a merged image **(C)** in the medial aspect of the ST. Single 0.5 μm sections displaying the localization (arrows) of relaxin-3 **(D)** synaptophysin **(E)** and a merged image **(F)** in the endopiriform nucleus. Single 0.5 μm section displaying localization (arrows) of relaxin-3 **(G)** synaptophysin **(H)** and a merged image **(I)** in the medial amygdala (anterior dorsal nucleus). Single 0.5 μm section displaying the localization (arrows) of relaxin-3 **(J)** synaptophysin **(K)** and a merged image **(L)** in the medial amygdala (posterior ventral nucleus). Calibration bars 5 μm.

Synaptophysin (Syn) immunofluorescent staining was used to confirm the presence of relaxin-3 synapses/terminations on amygdala neurons. These studies resulted in granular labeling surrounding neuronal soma, which appear as immuno-negative “dark holes”. Syn boutons were clearly observed in the first 5–10 μm below the section surface and within this region Syn immunofluorescent boutons averaged 400 nm in diameter. Clear colocalization of Syn with relaxin-3-immunoreactive boutons was observed in the medial nucleus of the ST (Figures 12A–C), DEn (Figures 12D–F), MeAD (Figures 12G–I) and MePV (Figures 12J–L). In order to undertake a quantitative estimation of the occurrence of double labeling (Syn/relaxin-3) in the amygdala, tracks of fibers were measured and the number of Syn boutons associated with the fiber was quantified (Table 3). On average between 5–30% of relaxin-3 fibers did not contain any Syn labeling. For the remaining relaxin-3 positive fibers, Syn boutons occurred in 0.1–2 boutons per 10 μm of relaxin-3 fiber.

**Table 3:** **Quantitative estimation of the colocalization of synaptophysin (syn) and relaxin-3 in the different nuclei of the amygdala**.

Amygdala nucleus	% of RLN3 fibers without colocalization with Syn botons mean ± SEM	Average number of Syn boutons per 10 μm of RLN3 fiber ± SEM
SLEA	45.1 ± 3.1	0.61 ± 0.15
CeA	18.3 ± 2.3	1.05 ± 0.34
MeA	21.4 ± 9.0	1.97 ± 0.26
La	10.8 ± 2.4	4.94 ± 3.30
BL	29.2 ± 5.9	2.05 ± 0.62
BM	17.4 ± 7.0	2.16 ± 1.37
ST	27.8 ± 7.9	0.14 ± 0.32
Cortical	16.1 ± 8.6	2.04 ± 1.15

## Discussion

Using the anterograde tracers, mR and BDA, we observed a NI projection to specific regions in the amygdala including the nuclei of the ST and the SLEA. NI fibers extend throughout the medial nuclei of the ST, medial aspects of the SLEA, and in the TA extend throughout the medial amygdala. Dispersed fibers were also located in a broad band along the basomedial nuclei. This pattern agrees with the general distribution of relaxin-3 fibers which are present within the medial ST and SLEA, the medial amygdala, and in a broad band covering the basomedial nuclei. In addition, groups of anterograde and relaxin-3 fibers invaded specific centers like the caudal VMLa. This suggests that a significant population of relaxin-3 fibers targeting the amygdala arise from the NI, although, a contribution of relaxin-3 fibers from relaxin-3 producing neurons in other areas, namely the pontine raphe nucleus, ventral periaqueductal gray and/or the area dorsal to the lateral substantia nigra (Tanaka et al., 2005; Ma et al., 2007), cannot be ruled out.

According to our data, there is a dense concentration of relaxin-3 fibers in the medial amygdala which also contains a high concentration of RXFP3 receptors. In contrast, the central nucleus that also contains a high concentration of RXFP3 (Ma et al., 2007; Smith et al., 2010; Lenglos et al., 2014) is largely devoid of relaxin-3. In the same way, while we have observed a high concentration of relaxin-3 fibers in the medial ST, RXFP3 receptors are concentrated in the lateral ST. Thus, while in the medial amygdala, fibers and receptors occupy the same location, there is a mismatch in the distributions in the central nucleus and the nuclei of the ST. A mismatch between fibers and the distribution of postsynaptic receptors has been described for the amygdala opioid system (Agnati et al., 1986) and volume transmission, whereby peptides can be delivered into extrasynaptic spaces and diffuse for a long distance (Thureson-Klein et al., 1986; Zhu et al., 1986) is a possible form of neural communication in the amygdala. Although relaxin-3 is present in synapses in the thalamus (Tanaka et al., 2005) and in the medial septum (Olucha-Bordonau et al., 2012), a match-mismatch between fibers and receptors has also been described for the cholecystokinin (CCK) transmission system. CCK fibers innervate the lateral central nucleus of the amygdala, which does not display CCK_2_ receptors, and the intercalated nuclei, which express CCK_2_ receptors, whereas the basolateral amygdala does not contain fibers but is rich in CCK_2_ receptors (Pérez de la Mora et al., 2007). CCK delivered in the central amygdala of rodents in the absence of danger is thought to activate CCK_2_ receptors of the basolateral amygdala and depolarize these neurons, leading to a maintained basal level of arousal (Pérez de la Mora et al., 2008). Thus, volume transmission is particularly adapted to neuropeptide neurotransmission signaling (Pérez de la Mora et al., 2007), as there are no peptide re-uptake mechanisms and inactivation of peptides strongly depends on extracellular peptidases, which limit their diffusion through the extracellular space (Davis and Konings, 1993), as reported for substance P in the spinal cord (Duggan et al., 1990, 1992). In this regard, relaxin-3 can be slowly degraded by the insulin degrading enzyme (Bennett et al., 2009) which is also expressed in the brain (Kuo et al., 1993).

In addition to the medial extended amygdala, relaxin-3 and NI-anterogradely labeled fibers were observed in central extended amygdala and in the lateral, basolateral and basomedial nuclei. It is well documented that amongst the lateral basal and central nuclei, sensory information is conveyed from the lateral to the central nucleus (Pitkänen et al., 1997), a process important for emotional processing and fear conditioning (Amorapanth et al., 2000; Nader et al., 2001). In this regard, we have demonstrated that electrolytic lesions of the NI impair within session extinction of fear conditioning (Pereira et al., 2013). Thus, putative relaxin-3 projections from the NI to the medial and ventrolateral aspects of the lateral amygdala and to the basolateral and basomedial nuclei could subserve a modulation of the extinction process by activation of RXFP3 within these areas and/or in the adjacent CeA.

Anatomical and functional comparisons reveal parallels between the central relaxin-3 and neuropeptide S (NPS) systems. NPS is expressed in neurons located in only a few brain areas—including in the pericoerulear area, in the Kölliker-Fuse nucleus, and in a small number of neurons within the dorsomedial hypothalamic nucleus (Xu et al., 2004; Pape et al., 2010; Clark et al., 2011), a similar distribution to that in humans (Adori et al., 2015). A major target of the NPS system is the endopiriform nucleus, which is also densely targeted by relaxin-3 fibers. NPS infusion into the endopiriform nucleus induced an increase in glutamatergic excitation assessed in *ex vivo* preparations that, in turn, resulted in a general decrease in GABAergic inhibition in the basal and lateral amygdala and an enhancement of spike activity in these nuclei. In a parallel fashion, infusion of NPS in this nucleus results in a reduction of freezing in contextual but not cued fear conditioning (Meis et al., 2008).

In the present studies, we have identified and detailed direct inputs of NI and relaxin-3 fibers to neurons expressing the calcium-binding proteins PV, CB-28kD and CR. Putative contacts have been demonstrated in the lateral and basal amygdala nuclei and were clearly evident at the border between LaVM and BLP, in the CeA, and in the AHi transition area. Specific types of inhibitory neurons characterized by their expression of particular calcium-binding proteins, have been shown to play a central role in fear processing (Ehrlich et al., 2009). For example, PV neurons of the basolateral nucleus differentially fire in association with an auditory cue or after footshock, suggesting they constitute, along with amygdaloid principal neurons, a dynamic disinhibitory microcircuit (Wolff et al., 2014).

PV neurons are also known to inhibit principal neurons of the lateral amygdala during noxious or salient stimuli (Chu et al., 2012; Bienvenu et al., 2015). In the current study, contacts between anterogradely-labeled NI fibers and PV neurons were observed in the lateral amygdala, suggesting a possible action on interneurons influencing the pathway from the lateral nucleus via the basal nucleus and finally targeting the central nucleus, thus providing a modulatory capability for altering fear acquisition and extinction. We also observed that the intercalated nuclei contain relaxin-3 fibers and these nuclei that surround the lateral nucleus receive direct projections from the prefrontal cortex which mediate the suppression of fear responses during the extinction process (Quirk et al., 2003; Paré et al., 2004). Thus, NI relaxin-3 projections to the intercalated nuclei may contribute to the modulation of the neural processes of fear extinction (Pereira et al., 2013) and further investigations are warranted, particularly in light of related functional impacts of relaxin-3/RXFP3 signaling in related circuits.

In this respect, there is growing evidence that the NI plays an important role in modulating arousal and cortical activation via actions on the septohippocampal system and changes in hippocampal theta rhythm (4–12 Hz oscillations detectable in the EEG; Brown and McKenna, 2015; Orzeł-Gryglewska et al., 2015). NI stimulation was initially demonstrated to increase hippocampal theta power and in contrast, NI lesion significantly attenuated hippocampal theta activity induced by stimulation of the *reticularis pontis oralis* (Nuñez et al., 2006). Similarly, increased hippocampal theta power was observed after infusion of an RXFP3-specific agonist into the septum, and infusion of an RXFP3-specific antagonist in the septum dose-dependently attenuated hippocampal theta power and spatial working memory in rats (Ma et al., 2009). Furthermore, we have reported a differential effect of corticotrophin-releasing hormone (CRH) on NI-relaxin-3 neurons in urethane-anesthetized rats, whereby NI-relaxin-3 neurons exhibited spontaneous firing that was phase-locked with hippocampal theta oscillations, whereas non-relaxin-3 neurons did not (Ma et al., 2013). Similarly, fear processing is related to changes in theta rhythm. During retrieval of fear memories, the amygdala and hippocampus display phase-locked activity at theta frequency (Paré et al., 2002; Seidenbecher et al., 2003). More recently, unit recordings from the infralimbic prefrontal cortex, the hippocampus CA1 and the lateral amygdala revealed that the pattern of theta oscillations correlated with individual behavioral responses during fear acquisition, retrieval and extinction (Lesting et al., 2011, 2013). Synchronization of theta oscillations between hippocampus and amygdala could in part depend on a common projection from the NI.

Central CRH infusion has been demonstrated to activate NI neurons expressing CRH-R1 (Bittencourt and Sawchenko, 2000; Van Pett et al., 2000), and stress activated neurons in the NI express relaxin-3 (Tanaka et al., 2005; Banerjee et al., 2010). Central infusion of an RXFP3 agonist has been found to have anxiolytic effects in adult rats (Ryan et al., 2013a) and mice (Zhang et al., 2015). Thus, these effects of RXFP3 signaling on anxiety-related mechanisms may involve the amygdala, although further studies are required in rat and mouse in appropriate models to further assess this possibility.

In addition to the putative function of relaxin-3/RXFP3 signaling in stress mechanisms, another function attributed to the NI relaxin-3 system is the modulation of feeding behavior. Intra-hypothalamic or icv infusion of relaxin-3 or RXFP3-selective agonists results in promotion of feeding in satiated rats (McGowan et al., 2005, 2006; Haugaard-Kedström et al., 2011; Shabanpoor et al., 2012) and some effects on feeding behavior may occur via actions within the amygdala. Rats in which obesity was induced with a high fat diet and refeeding after food deprivation, displayed increased expression of RXFP3 in the central amygdala, as well as in the paraventricular hypothalamic nucleus, NI and nucleus of the olfactory tract, whereas such changes were not observed in rats in which a high fat diet did not induce obesity (Lenglos et al., 2014).

Finally, recent research has shown that central (icv) administration of an RXFP3 antagonist produced a dose-related decrease in self-administration of alcohol (Ryan et al., 2013b). This effect is in part mediated by effects within the extended amygdala, as bilateral infusion of an RXFP3 antagonist into the bed nucleus of the ST decreased self-administration of alcohol and stress-induced reinstatement of alcohol seeking (Ryan et al., 2013b). Thus the NI/relaxin-3 innervation of the medial divisions of the ST identified in the current study may provide the relaxin-3 input involved.

In general, it appears that relaxin-3 positive fibers that can potentially release the peptide are present in the medial division of the amygdala and medial nuclei of the ST, while neurons which express RXFP3 mRNA are present in these areas, but are more concentrated in the central amygdala and the lateral nuclei of the ST. Indeed a mismatch between areas expressing peptide receptors and the location of fibers delivering peptide ligand is also observed with the CRH innervation of the amygdala. While the lateral central amygdala receives a dense innervation of CRH fibers (Swanson et al., 1983; Sakanaka et al., 1986, 1987); neurons expressing the CRH-R1 receptor are concentrated in the medial nucleus of the central amygdala (Van Pett et al., 2000). However, in transgenic mice expressing CRH-R1, the dendritic tree of CRH-R1 neurons may be in close contact with CRH fibers within surrounding areas (Justice et al., 2008). Therefore, at this stage, it is possible that synaptic, extra-synaptic or volume transmission mechanisms may be involved in peptidergic transmission within the medial and lateral divisions of the ST or within the medial and central nuclei of the amygdala.

In conclusion, we have documented the topographical projections from the NI to selective regions (nuclei/subnuclei) of the amygdala, including the medial and extended amygdala, the lateral and basal nuclei and the endopiriform nucleus, which may modulate various amygdala-driven functions, including feeding, social behavior, fear conditioning and extinction, and reward and motivation. Separate or collateral projections of NI neurons to the septum, hippocampus and amygdala may provide a neural network capable of rhythmic theta synchronization between the hippocampus and the amygdala during emotionally-related processes. The presence of relaxin-3 in a relevant population of these NI neurons and pharmacological studies with RXFP3-selective peptides suggest that relaxin-3/RXFP3 signaling may contribute to these important physiological and behavioral modalities.

## Author Contributions

FNS and CWP performed double ICC and camera lucida drawings, AMS-P, performed double immunofluorescence, prepared figures and reported intellectual input, MO performed double IF for Syn and CBP, SM and ALG provided intellectual input and discussed along the various versions, FEO-B performed tracing injections, double ICC and IF, wrote the first draft, corrected versions and prepared figures.

## Conflict of Interest Statement

The authors declare that the research was conducted in the absence of any commercial or financial relationships that could be construed as a potential conflict of interest.

## Footnotes

^1^The bed nucleus of the stria terminalis has been classically abbreviated as BST or BNST, however the different sub-subdivisions of this complex make this unsuitable, thus following the 7th Edition of the Paxinos and Watson Atlas of the Rat Brain, we have adopted the simplified abbreviation of ST to refer to these nuclei.
